# Cellulose Nanocrystals Obtained from *Agave tequilana* Weber Bagasse as Reinforcing Agents for CPC-30R Portland Cement Mortar

**DOI:** 10.3390/nano16181142

**Published:** 2026-09-11

**Authors:** Manuel Alberto Gallardo-Sánchez, Gabriel Landázuri-Gómez, José Anzaldo-Hernández, Salvador García-Enriquez, José Manuel Vicente Gómez Soberón, Emma Rebeca Macías-Balleza

**Affiliations:** 1 Department of Civil Engineering and Topography, University of Guadalajara, Guadalajara 44430, Mexico; manuel.gallardo@academicos.udg.mx; 2 Department of Chemical Engineering, University of Guadalajara, Guadalajara 44430, Mexico; gabriel.landazuri@academicos.udg.mx; 3 Department of Wood Cellulose and Paper, University of Guadalajara, Guadalajara 44430, Mexico; jose.anzaldo@academicos.udg.mx (J.A.-H.); salvador.genriquez@academicos.udg.mx (S.G.-E.); 4 Department of Construction Technology, Barcelona School of Building Construction, Polytechnic University of Catalonia, Av. Doctor Marañón 44-50, 08028 Barcelona, Spain

**Keywords:** cellulose, nanocrystals, mortars, construction, sustainability

## Abstract

Cellulose nanocrystals (CNCs) have been widely investigated as reinforcing agents in different matrices because of their unique properties, including high specific mechanical properties, biocompatibility, and durability. CNCs can be obtained from lignocellulosic residues such as *Agave tequilana* bagasse, a byproduct of the tequila industry. In this study, cementitious mortar matrices were reinforced with CNCs produced by acid hydrolysis using sulfuric acid (CNC S) and hydrochloric acid (CNC H). Mortar containing different CNC contents, expressed relative to cement mass, was prepared and evaluated in terms of compressive and flexural strength. The resulting materials were further characterized by thermogravimetric analysis (TGA), scanning electron microscopy (SEM), X-ray diffraction (XRD), and Fourier-transform infrared spectroscopy (FTIR). CNC incorporation increased both compressive and flexural strength, with the best-performing formulations containing 0.35 wt.% CNC S and 0.5 wt.% CNC H. SEM observations showed fewer and smaller microcracks and a narrower interfacial transition zone (ITZ) in CNC-containing mortars than in the control. Thermal and structural analyses indicated that CNC incorporation modifies cement hydration and the organization of hydrated phases. FTIR spectra also showed changes in bands associated with hydroxyl, sulfate, carbonate, and silicate environments, suggesting different interactions of CNC S and CNC H with the cementitious matrix. Overall, Agave-derived CNCs show potential as low-dosage reinforcing additives for Portland cement mortars while providing a value-added route for agroindustrial waste.

## 1. Introduction

In recent decades, there has been a growing demand for eco-friendly and sustainable products made from waste materials [1]. This has required increased research and development efforts to recycle waste materials into biodegradable products with low environmental impact [2,3,4,5].

Cellulose was first identified by the French chemist Anselme Payen in 1838, who isolated cellulose from various plants and determined its molecular formula (C_6_H_10_O_5_) through elemental analysis [6]. Plants are composed of interlocking cells forming layers in which cellulose serves as the primary structural component and provides strength to the plant structure [7]. It is also present in living organisms other than plants, such as bacteria and tunicates [8].

Cellulose nanocrystals (CNCs) are nanoscale cellulose particles with a highly crystalline, typically rod-like morphology [9]. They can be produced by controlled acid hydrolysis and generally exhibit nanometric diameters and lengths of hundreds of nanometers, depending on the cellulose source and processing conditions [10]. Their high stiffness, surface functionality, and nanoscale dimensions make them attractive reinforcing agents for a variety of matrices, including cement-based materials such as cement paste, mortar, and concrete.

Cement is the most widely used construction material in the world. It provides useful and desirable properties, such as compressive strength (it is the construction material with the highest strength per unit cost), durability, and aesthetic appeal for a wide range of construction applications [11]. The reaction by which cement transforms into a binding agent occurs when water is added to it. The silicates and aluminates present in cement form hydration products, such as calcium silicate hydrate (C_3_S_2_H_3_), known as C-S-H gel, in which the released lime precipitates as calcium hydroxide [Ca(OH)_2_] and forms small crystals that, over time, produce a firm, hard mass known as hydrated cement [12,13].

In an effort to improve the properties of the cementitious matrix, research has been conducted on the addition of wood particles [14,15,16], wood extracts [17], cellulose fibers [18,19,20,21,22,23,24], modified cellulose fibers [25,26], cellulose pulp [27,28,29], carbon nanotubes and nanofibers [30,31], microcrystalline cellulose particles [3], cellulose nanofibers [24,32,33], and cellulose nanocrystals [34,35,36,37,38].

Fu et al. (2017) added 0.2, 0.5, 1.5, and 2% CNC to cement pastes, obtaining higher flexural strength than the control samples [36]. Parveen et al. (2018) found that MCC at 0.5% and 1% increased flexural strength by 19% and 16%, respectively, and compressive strength by 51% for both [37]. They also reported a lower number of pores compared to the reference mortar, indicating that MCC helps improve the homogenization of the cementitious matrix, thereby increasing its stiffness. More recently, Ai et al. (2024) reported that the incorporation of CNCs into cement pastes can influence hydration and mechanical performance depending on the curing time [38]. Although CNCs initially delayed cement hydration, their reinforcing effect became more evident at later ages. At 3 days, the addition of CNCs increased flexural strength by approximately 23% compared with the control, while an improvement of 16.6% was maintained after 28 days. The authors attributed this behavior to the nano-needle morphology of CNCs, which can interact with microcracks and form a nanoscale reinforcing network that hinders crack propagation, together with an increased degree of hydration at later stages. Firdissa et al. (2025) investigated the incorporation of CNCs obtained from sugarcane bagasse as a partial replacement for ordinary Portland cement [39]. The authors reported that a 10% CNC replacement produced the highest compressive strength (42.4 MPa) after 28 days, representing a 13.9% increase compared with the control (37.2 MPa). The incorporation of CNCs also affected the setting behavior and reduced the unit weight of the concrete, although CNC contents above the optimum level resulted in decreased mechanical performance, highlighting the importance of controlling CNC dosage in cementitious composites. Feng et al. (2025) investigated the effect of CNCs on the mechanical properties and chloride resistance of cement pastes [40]. The optimal CNC contents were 0.1% and 0.2% for water-to-cement ratios of 0.3 and 0.5, respectively. At these concentrations, CNC incorporation increased compressive strength by 18.8–22.14%, splitting tensile strength by 19.4–26.38%, and flexural strength by 32–44.67%. Moreover, CNC addition reduced the mechanical strength loss caused by chloride exposure, suggesting that CNCs can simultaneously improve the mechanical performance and durability of cementitious materials.

Zheng et al. (2023) investigated the hydration characteristics of C_3_S by mixing it with different amounts of CNC and examining the chemical environment of the hydration products [41]. They reported that CNC has an effect like that of superplasticizers in reducing exothermic hydration. They also found that CNC promotes the growth of Ca(OH)_2_ and increases the grain size of Ca(OH)_2_. CNCs provide additional nucleation sites, which reduce the degree of polymerization and shorten the main chain length of the resulting C–S–H gel. A separate hydration test confirmed that the CNCs sequentially adsorbed calcium ions and silicate ions, which promoted the formation of a C–S–H gel layer on the surface of the CNCs. The size of the nanocrystals allows them to fill the pores that form in the matrix microstructure, reducing the percentage of porosity; it also slows the hydration heat, which increases water accessibility and, therefore, the C–S–H gel content. The presence of nanocrystals eliminates the microcracks that form early in the curing process (0 to 7 days), which also imparts stiffness [24,35]. In turn, these CNCs are compatible with the C–S–H matrix gel interface, promoting good coupling between the two [42].

Montes et al. (2020) reported that the rheological behavior of cement pastes containing CNCs was not strongly correlated with the zeta potential, the average particle length of the CNCs, or the average aspect ratio of the CNC particles [43]. However, it was found that the maximum reduction in the elastic limit occurred at higher CNC dosages as the water-cement ratio increased. Furthermore, rheological measurements suggested that at lower dosages (<0.2%), CNC behaved more like a water-reducing additive, reducing the elastic limit by up to 54%, whereas at higher doses (more than 0.5%), CNCs increased the paste’s elastic limit, similar to certain viscosity-modifying additives.

In this study, cellulose nanocrystals obtained from *Agave tequilana* bagasse, an agroindustrial residue generated by tequila production in western Mexico, were evaluated as reinforcing agents for Portland cement mortars prepared in accordance with ASTM C348-21 [44]. Their effects on compressive and flexural strength were assessed, and the resulting materials were characterized by scanning electron microscopy (SEM), thermogravimetric analysis (TGA), X-ray diffraction (XRD), and Fourier-transform infrared spectroscopy (FTIR). The valorization of agave bagasse through CNC production provides an opportunity to convert a regionally abundant agroindustrial residue into a value-added material for more sustainable cementitious composites.

## 2. Materials and Methods

CNCs derived from Agave tequilana bagasse were used, obtained using the procedure described by Gallardo et al., 2021 [45]: 0.1 N standardized sodium thiosulfate (Na_2_S_2_O_3_) solution, 0.1 N standardized potassium permanganate (KMnO_4_) solution from Golden Bell (Zapopan, Mexico); sodium hydroxide (NaOH, 97% purity), potassium dichromate (K_2_Cr_2_O_7_, 99% purity) from Karal S.A. de C.V. (León, Mexico); sodium chlorite (NaClO_2_, 80% purity), sulfuric acid (H_2_SO_4_, 97% purity), hydrochloric acid (HCl, 37% purity), anthraquinone (C_14_H_8_O_2_, 97% purity), hydrogen peroxide (H_2_O_2_, 30% purity), and 1.0 M cupriethylenediamine solution (Cu(H_2_NCH_2_CH_2_NH_2_)_2_ (OH)_2_) from Sigma Aldrich (Toluca, Mexico); potassium iodide (KI, 99% purity) from Jalmek (San Nicolás de los Garza, Mexico). Portland cement CPC-30R from Cementos Fortaleza (Hidalgo, Mexico) was used to manufacture the mortars. Ottawa silica sand 16-100 (ASTM C 109 [46], C778 H 3825 [47]) from ELVEC (Mexico City, Mexico) was also used.

### 2.1. Obtaining Soluble-Grade Pulp

Soluble-grade pulp was obtained according to the procedure reported by Gallardo et al., 2019 [48]. The procedure consists of placing 1 kg of bagasse, previously de-medullated and washed, in a reactor (Jayme-type Digester, model R25/3) in a solution of NaOH and anthraquinone for 2 h at 160 °C. The resulting pulp was characterized according to standard TAPPI methods: the Kappa number was determined using TAPPI Standard 236 om-22 [49]; viscosity and degree of polymerization were determined according to TAPPI Standard T230 om-19 [50]; and α, β, and γ cellulose were determined according to TAPPI Standard T203 cm-22 [51]. Subsequently, the pulp underwent a bleaching process as reported by Gallardo et al., 2019 [48], which consists of five stages: (1) oxygen stage (O), (2) chlorine dioxide first stage (DO), (3) oxygen-peroxide-reinforced stage, (4) chlorine dioxide final stage, and (5) oxygen-reinforced peroxide stage. The pulp obtained at the end of these stages was washed, centrifuged, and stored at 4 °C for later use.

### 2.2. Preparation of Cellulose Nanocrystals via Acid Hydrolysis

To prepare CNC, the procedure reported by Gallardo et al. (2021) [45] was used, starting with soluble-grade pulp, which was subjected to acid hydrolysis with H_2_SO_4_ and HCl, the former under conditions of 64% acid concentration, 60 °C temperature, and a reaction time of 70 min. The resulting CNCs are identified as CNC S, whereas hydrolysis with HCl was carried out at a concentration of 8 N, 90 °C, and for 200 min; the resulting nanocrystals are identified as CNC H. In both cases, after hydrolysis, a dialysis process is carried out with HPLC-grade water with a resistivity of 18.2 MOhm × cm at 25 °C, the water was obtained using a Simplicity® UV water purification system (Merck Millipore, Molsheim, France) and the dialysis is performed until a pH between 5 and 5.5 is reached. The resulting suspension was then passed through 2, 1.5, and 1 µm filters. The concentration of the CNC solution was determined by gravimetry, and these were subsequently used in aqueous solutions.

### 2.3. Preparation of the Mortar Mixtures

Mortar mixtures were prepared as specified in ASTM C348-21 [44]. The concentrations of CNC S and CNC H in the mortars were 0.1, 0.2, 0.35, 0.5, and 1.0% by weight of cement, in accordance with the values reported in the literature [35,39,52]. To do this, the aqueous CNC solution was added, and the volume of water was subtracted from the total volume of the mixture. For each CNC concentration, 10 test specimens (5 cubes and 5 beams) were prepared in accordance with ASTM C109/C109M-21 [46] and ASTM C348-21 [44], which specify that the dimensions of the cubes and beams must be 5 × 5 × 5 cm^3^ and 4 × 4 × 16 cm^3^, respectively. Cubes are used to evaluate compressive strength, and beams are used to evaluate flexural strength. Each specimen (cube/beam) is removed from the mold 24 h after being cast and immersed in lime water for 28 days. After this period, their strength (compressive and flexural) is evaluated, and they are characterized using scanning electron microscopy (SEM), thermogravimetric analysis (TGA), X-ray diffraction (XRD), and Fourier-transform infrared spectroscopy (FTIR).

### 2.4. Characterization of the Mortars

#### 2.4.1. Compressive Strength

The compressive strength of the cubes was determined using a 25,000 kg universal testing machine manufactured by American Machine and Metals Inc., model RIEHLE (East Moline, IL, USA), and the tests were conducted in accordance with ASTM C109/21 [46]. A load was applied to the cubes at a rate of 900 to 1800 N/second until the specimen failed.

#### 2.4.2. Flexural Strength

The flexural strength of the beams was determined using a PRÜFTECHNIK CADIS universal testing machine with a capacity of 5000 kg, and the tests were conducted in accordance with ASTM C348-21 [44]. The beams were placed on two support points 12 cm apart, with the upper support located exactly at the midpoint of the beam. A load was applied at a rate of 2600 N/min until the beam failed. The flexural strength in MPa was obtained with $S_{F}=0.0028\;P$, where P is the total maximum load in N.

#### 2.4.3. Analysis of the Matrix Microstructure by Scanning Electron Microscopy (SEM)

A scanning electron microscope (SEM) was used to analyze the microstructure of the mortars; the instrument was a JEOL model JMS-6510 (JEOL Ltd., Tokyo, Japan), equipped with a GATAN ALTO-1000 cryo-probing unit, manufactured by Gatan (UK), an EDS detector, and backscattered electron detection, operating at 20 kV, with a current of 127 μA and a working distance of 15 mm. Two imaging modes were used: secondary electron imaging (SEI) and backscattered electron imaging (BEI). For this analysis, the untested cubes were taken and cut to obtain a small 1 cm^3^ cube from the interior of the sample, which was then dried in an oven at 40 °C for 24 h. They were then placed on Epofix resin with a catalyst to form a pellet, left under vacuum for 5 to 10 min, and placed in an oven at 40 °C for 8 h to allow the resin to solidify. The pellets were polished using P220 and P500 sandpaper and a glass plate with Al_2_O_3_ having a particle size of 12 μm. The samples were placed in an ultrasonic bath to remove the alumina, and metallographic polishing was performed using 1 μm diamond paste for 10 min and 0.25 μm diamond paste for 10 min on velvet. Finally, a carbon coating was applied to the polished surface for analysis by SEM. The resulting micrographs were analyzed using LabVIEW’s VISION 2018 software.

#### 2.4.4. Analysis of the Matrix’s Degree of Hydration by Thermogravimetric Analysis (TGA)

A thermogravimetric analysis (TGA) system manufactured at the Polytechnic University of Catalonia (UPC) was used. It consists of a Naber D-25DFL furnace and a PCE LSM200 balance with an accuracy of 0.0001 g; these are connected to a computer running specialized software for the system developed by the UPC. The residue left over from cutting the samples for SEM is used in TGA. It is ground in a mortar and passed through a 4 mm sieve; the retained material is removed, and the remainder is placed in an oven at 40 °C for 24 h. The sample is then sieved through 4, 2, 1, 0.5, 0.25, 0.125, and 0.063 mm sieves; the material retained on each sieve is weighed, and its percentage of the total weight of the sieved sample is calculated. A representative sample totaling 8 g is prepared, in accordance with the weight percentages calculated for each sample. The sample was placed in the test crucible and analyzed up to 950 °C using a nominal heating rate of 3 °C min^−1^. Sample mass and temperature were automatically recorded throughout the analysis, typically at 10 s intervals. One representative TGA run was performed for each mortar formulation.

#### 2.4.5. Analysis of Plane Orientations and Crystallinity by X-Ray Diffraction (XRD)

The equipment used was an Empyrean diffractometer manufactured by PANanalytical (Worcestershire, UK), operating at 45 kV and 40 mA, with an incident angle ranging from 5 to 70°, in 0.2° increments, and a dwell time of 30 s per step, using CuKɑ radiation filtered through a Ni filter and a graphite monochromator. The powdered sample was placed in a sample holder up to the indicated mark, and the analysis was performed. The results were analyzed using OriginPro 2021 (OriginLab Corporation, Northampton, MA, USA).

#### 2.4.6. Analysis of the Molecular Structure of the Matrix by Fourier-Transform Infrared Spectroscopy (FTIR)

The molecular structure of the mortar samples was analyzed by Fourier-transform infrared (FTIR) spectroscopy using a PerkinElmer Spectrum GX spectrophotometer (Norwalk, CT, USA). Spectra were collected from 4000 to 400 cm^−1^ at a resolution of 4 cm^−1^ by averaging 16 scans. The samples were ground in a mortar, mixed with KBr, and pressed into pellets for analysis.

## 3. Results and Discussion

The characteristics of the soluble-grade pulp used to produce the CNCs correspond to those previously reported by Gallardo et al. (2019) [48], with a KN of 23 ± 3, a viscosity of 10.18 cp, a DP of 750, 94 ± 3% α-cellulose, and a crystallinity value of 79.2%. The main physicochemical characteristics of the CNCs, previously reported by Gallardo et al. (2021) [45], are summarized in Table 1. CNC S exhibited slightly higher crystallinity and smaller dimensions, aspect ratio, and hydrodynamic diameter than CNC H. In particular, CNC H exhibited a higher width-to-height (W/H) aspect ratio than CNC S, consistent with the aggregation observed in the previously reported AFM images [45]. The two CNCs also differed in their surface chemistry: CNC S contained 166 mmol kg^−1^ of total sulfate groups as a consequence of H_2_SO_4_ hydrolysis, whereas CNC H did not contain significant surface sulfate groups. These differences in dimensions and surface functionality are relevant for interpreting their behavior in the cementitious matrix.

### 3.1. Compressive and Flexural Strength

**Figure 1:**
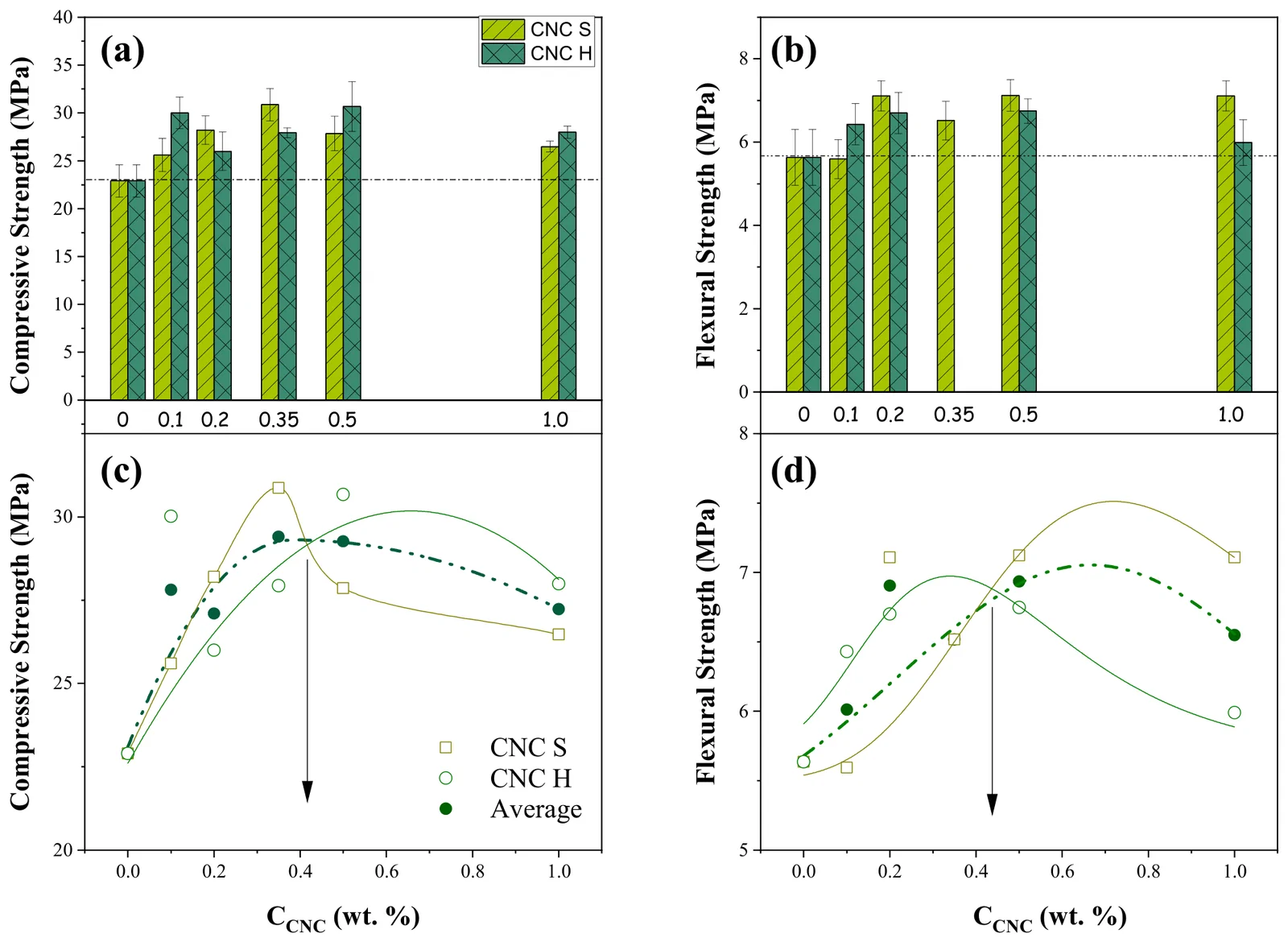
Compressive strength (**a**,**c**) and flexural strength (**b**,**d**) of mortars as a function of CNC content. Hollow symbols represent the experimental data for CNC S and CNC H; full symbols correspond to the average of both types of CNC; solid and dotted lines represent the fitted curves.

Figure 1a and Figure 1b show the effect of CNC S and CNC H content on the compressive and flexural strengths of mortar specimens, respectively, after 28 days of curing. The corresponding values and statistical analysis are provided in Table S1 of the Supplementary Materials. Sample codes consist of the letter M followed by the CNC concentration (0, 0.1, 0.2, 0.35, 0.5, or 1 wt.%) and a final letter indicating the hydrolysis method: S for sulfuric acid and H for hydrochloric acid.

For compressive strength (Figure 1a), the maximum values were obtained at 0.35 and 0.5 wt.% for mortars containing CNC S and CNC H, respectively, reaching 30.9 ± 1.7 and 30.7 ± 2.6 MPa. The CNC-free reference sample (M0) exhibited a compressive strength of 22.9 ± 1.7 MPa; therefore, CNC incorporation increased compressive strength by up to approximately 35% for CNC S and 34% for CNC H. Previous studies have reported a 20% increase in compressive strength after the addition of cellulose nanofibers (CNFs) [33], whereas the incorporation of 3 wt.% microcrystalline cellulose (MCC) produced values 7–13% lower than those of the reference sample [3]. Mazlan et al. (2016) [42] used CNC obtained from commercial cellulose to prepare 125 cm^3^ mortar cubes with a water-to-cement ratio of 0.5 and reported an increase in compressive strength of up to 38% at 0.2% CNC after 28 days. In that study, compressive strength increased at all CNC concentrations, in agreement with the general trend observed here.

Flexural strength showed a similar trend (Figure 1b). The maximum value for CNC S was 7.1 ± 0.4 MPa at 0.5 wt.% (M0.5 S), whereas CNC H reached 6.7 ± 0.3 MPa at the same concentration (M0.5 H). The unmodified mortar exhibited a flexural strength of 5.6 ± 0.7 MPa, corresponding to increases of approximately 27% and 20% for CNC S and CNC H, respectively.

Similar results have been obtained in cement pastes using MCC and CNC derived from wood and extracted by hydrolysis with H_2_SO_4_, as reported by Parveen et al. (2018) [37], who incorporated MCC into cement at 0.5, 1, 1.5, 2, and 2.5 wt.% relative to cement and reported increases in flexural strength of 19% and 16% at 0.5 and 1 wt.%, respectively. The same study also found a lower number of pores compared to the reference mortar, indicating that MCC helps improve the homogenization of the cementitious matrix, thereby increasing its stiffness. Fu et al. (2017) [36] added CNC to cement pastes at concentrations of 0.2, 0.5, 1.5, and 2%, obtaining flexural strengths measured by the B3B method that were 20% higher than those of the control samples. Cao et al. (2016) [35] prepared Type V cement pastes with a water-to-cement ratio (w/c) of 0.35 and cement-to-volume (*w*/*v*) percentages of CNC of 0.04, 0.1, 0.2, 0.5, 1, and 1.5%. Flexural strength was evaluated using the B3B method, and values 50% higher than those of the control sample were obtained when 1% replacement was used after 28 days of curing. Similarly, where CNF has been used, increases of 15% have been reported [33].

The measurements yield similar results for both additives (CNC S and CNC H). Furthermore, statistical analysis (see Table S1 of Supplementary Materials) shows that, for compressive strength, the type of additive (CNC S or CNC H) does not result in a significant difference, with *p* > 0.05 in both cases. In Figure 1c, the response for each of the analyzed systems is plotted separately as a function of the CNC concentration for mortar containing CNC S and CNC H, as well as the average of both types of CNC (full symbols). The solid and dotted lines represent the best fit to the following equation:

(1) $$y=y_{0}+Ae^{\left({(-e}^{\left(-z\right)}-z+1\right)}$$

where *z* = (x − xc)/w, y_0_ is the offset of y, A is the amplitude, xc is the center, and w is the width of the curve. The fitting parameters are shown in Table 2. In this graph, it can be seen that although the statistical analysis shows no significant difference between the types of CNC (Table S1 in the Supplementary Materials), when analyzed individually, there is a crossover point at a concentration of approximately 0.4 wt.% CNC; for concentrations below 0.4, the specimens reinforced with CNC S exhibit greater compressive strength than those reinforced with CNC H, and above this concentration, the behavior is reversed. On the other hand, with regard to flexural strength (Figure 1d), statistical analysis shows that there is a dependence on the type of additive—CNC S or CNC H (Table S1, Supplementary Materials)—and that at concentrations below 0.4% CNC, mortars reinforced with CNC H exhibit higher values than those reinforced with CNC S; the opposite is true at higher concentrations. It is also evident that at this crossover point, when comparing the two properties side by side, at concentrations below 0.4% CNC, CNC S exhibits higher compressive strength but lower flexural strength; at concentrations above 0.4%, CNC H exhibits higher compressive strength but lower flexural strength.

The strength improvements obtained in this study were achieved at relatively low CNC contents. Both compressive and flexural strength reached maxima between 0.35 and 0.5 wt.% CNC. Although strength decreased between the optimum concentration and 1 wt.% CNC, the values remained above those of the reference mortar (M0). For compressive strength, the optimum CNC content depended on CNC type, with maximum values obtained at 0.35 wt.% for CNC S and 0.5 wt.% for CNC H. In contrast, maximum flexural strength was obtained at 0.5 wt.% for both CNC types. This behavior suggests that the optimum CNC dosage depends not only on CNC physicochemical characteristics but also on the mechanical response considered. Differences in particle dimensions, surface functionality, and aggregation behavior may influence CNC dispersion and CNC–matrix interactions, particularly under compressive loading. Changes in hydration kinetics and the development of C–S–H may also contribute to the non-monotonic mechanical response [42,53,54]. At higher CNC contents, excessive interactions among nanocrystals may promote agglomeration and the formation of weak interfaces or local stress-concentration regions, thereby reducing the reinforcing efficiency [33,54,55].

The main finding of the mechanical analysis is that substantial improvements in compressive and flexural strength were achieved at relatively low CNC contents. These improvements may be associated with the water-retention capacity of CNCs and their ability to gradually release water during cement hydration [56,57], thereby favoring the development of C–S–H gel [42]. Their nanoscale dimensions may also allow CNCs to occupy small voids within the cementitious microstructure. In addition, the high stiffness of CNCs (reported elastic modulus of approximately 110–220 GPa) [34] may contribute to mechanical reinforcement when effective stress transfer occurs at the CNC–matrix interface. The interfacial transition zone (ITZ) between sand grains and the cement paste is particularly relevant because microcracks frequently initiate in this region [34]; therefore, the ITZ was examined by SEM to evaluate possible microstructural changes associated with CNC incorporation.

Although all mortars were prepared at a constant water-to-cement ratio and under the same experimental conditions, fresh-state properties such as flowability, consistency, and water-reducing admixture demand were not evaluated. Considering the high specific surface area and hydroxyl-rich surface of CNCs, their incorporation may affect water demand and mortar rheology. Therefore, a possible contribution of fresh-state properties to the observed mechanical behavior cannot be excluded and should be considered in future studies.

### 3.2. Scanning Electron Microscopy (SEM)

**Figure 2:**
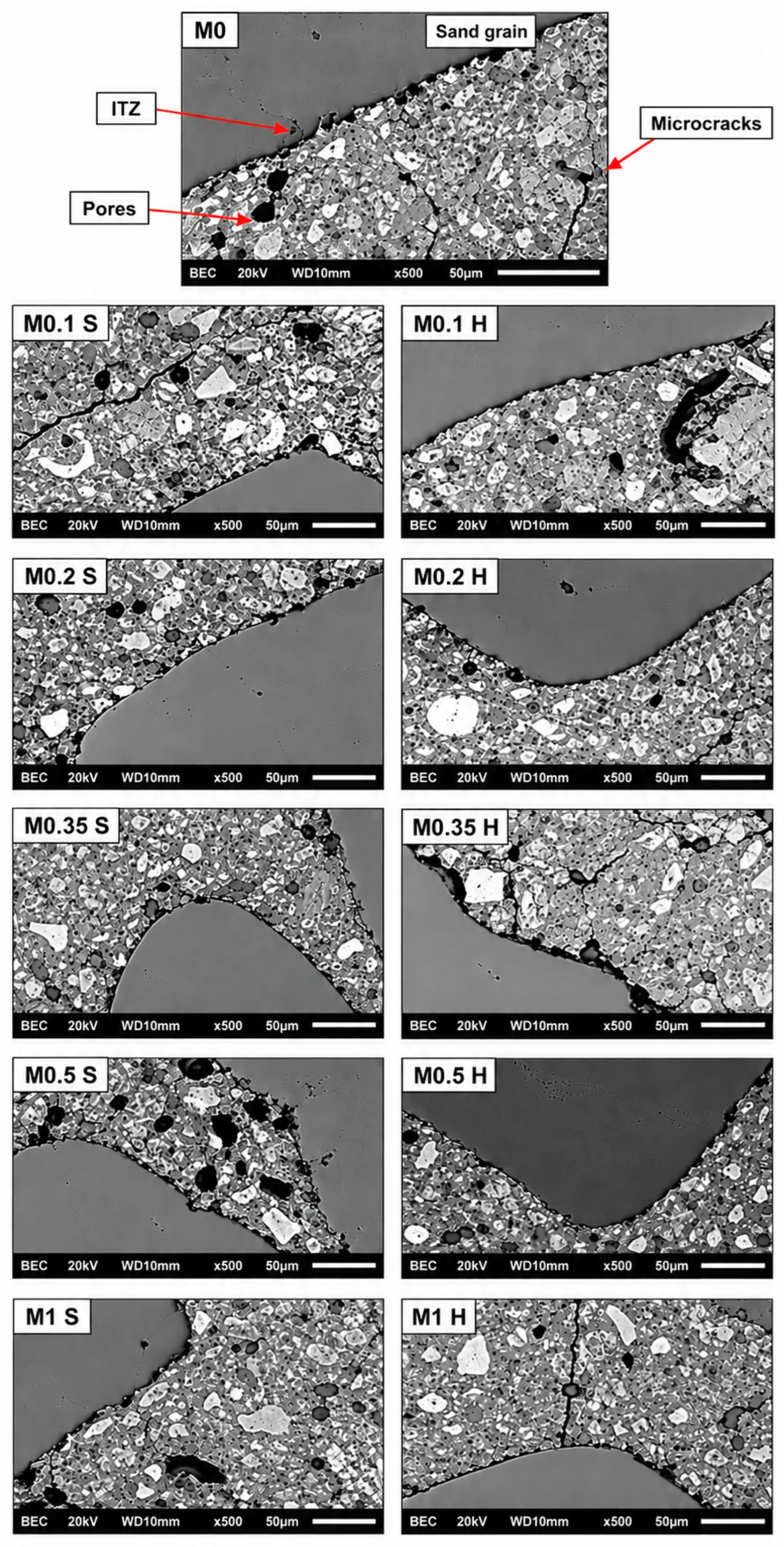
Representative SEM micrographs of mortars containing CNC S and CNC H at different CNC concentrations. Selected microstructural features, including microcracks and the interfacial transition zone (ITZ), are indicated.

Figure 2 shows SEM micrographs of the analyzed mortars. The main microstructural features identified include the cementitious matrix, sand grains (darker regions), the interfacial transition zone (ITZ) between the cement paste and sand grains, unhydrated cement particles (bright regions), calcium-rich crystalline phases (white regions), capillary pores (dark regions), and hydration products (gray regions).

The M0 control sample exhibits a relatively thick ITZ, approximately 4 μm, together with numerous microcracks of different sizes. These microcracks may arise from two main sources: sample preparation for SEM analysis and shrinkage of the cementitious matrix during setting and curing [58].

**Figure 3:**
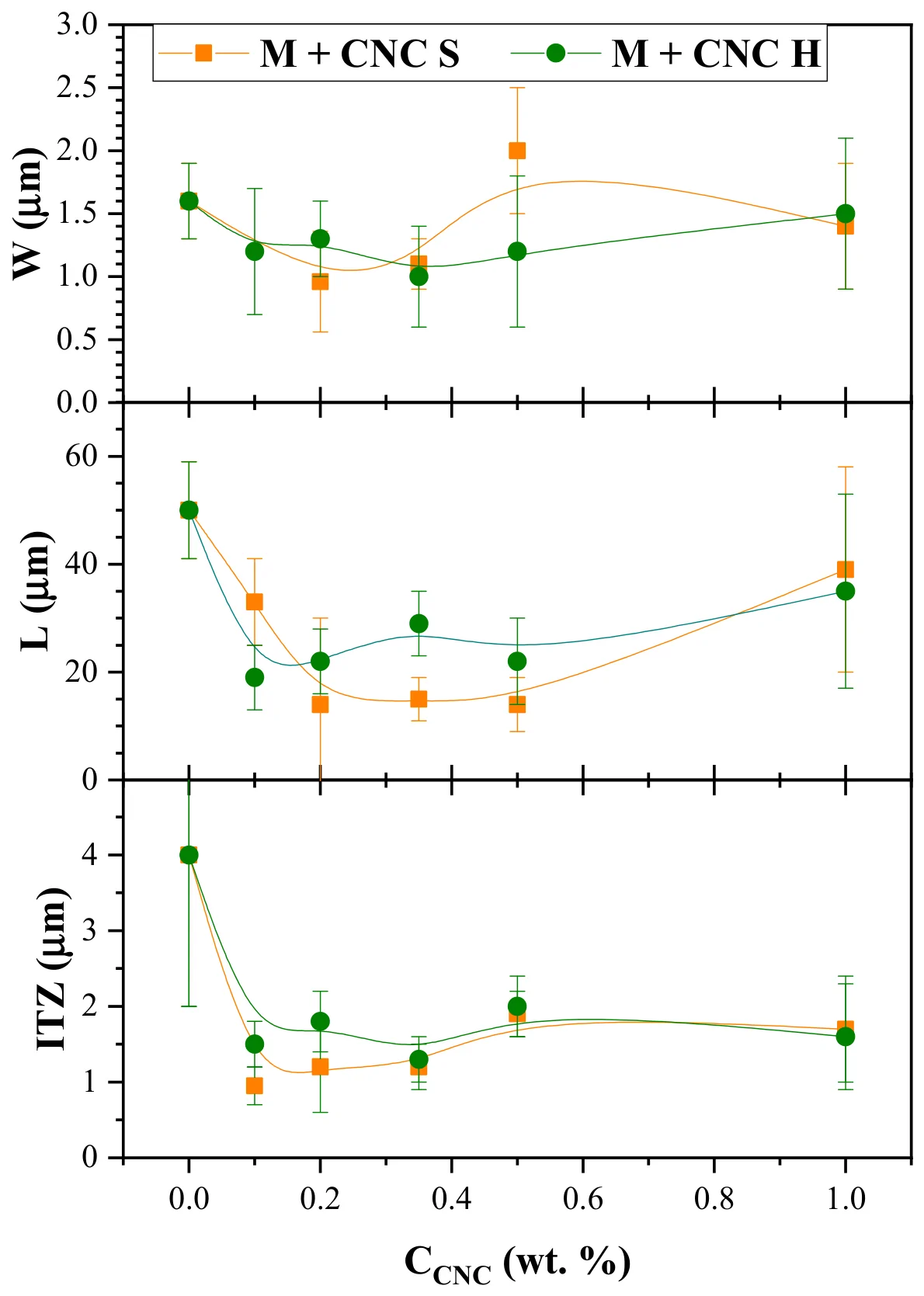
Average microcrack width (W), microcrack length (L), and interfacial transition zone (ITZ) thickness of mortars containing CNC S and CNC H as a function of CNC content. Values represent mean ± standard deviation.

In contrast, the M0.35 S and M0.5 H samples exhibit a more homogeneous microstructure, with fewer and smaller microcracks than the M0 sample, consistent with previous reports [3,59,60]. The ITZ is also considerably narrower, with a thickness of approximately 1–1.1 μm. Figure 3 summarizes the average width and length of the microcracks and the ITZ thickness determined from the SEM micrographs. Multiple measurements of microcrack width, microcrack length, and ITZ thickness were performed for each mortar formulation using ImageJ version 1.51 2017 (Wayne Rasband, National Institutes of Health, Bethesda, MD, USA), and the results are expressed as mean ± standard deviation. The corresponding quantitative values are reported in Table S2 of the Supplementary Materials.

As the CNC content increases, both the microcrack dimensions and ITZ thickness decrease until reaching minimum values at CNC concentrations close to those yielding the highest compressive strengths for both CNC S and CNC H. Above the optimum CNC content, the dimensions of the microcracks and the ITZ tend to increase again. This behavior is consistent with the mechanical results and suggests that, at appropriate concentrations, CNC incorporation promotes a more homogeneous microstructure and improves the quality of the cement paste–sand interface. The reduction in microcrack dimensions and ITZ thickness may therefore contribute to the enhanced mechanical performance of the CNC-containing mortars.

The hydrophilic nature of CNCs, arising from the abundance of surface hydroxyl groups, provides them with a high affinity for water and may influence water distribution within the cementitious matrix. CNC surfaces may also interact with cement hydration products through hydrogen bonding and other interfacial interactions [3]. Their ability to retain water and subsequently make it available locally during hydration may favor the formation of hydration products, including C-S-H, in the vicinity of the CNC surface. In addition, CNCs may act as nucleation sites for hydration products, contributing to the development of a denser and more homogeneous microstructure. The morphology of C-S-H and the porosity of cementitious materials are strongly influenced by cement composition and, particularly, by the water-to-cement ratio (w/c) [61,62,63]. The chemical structure of cellulose causes it to expand significantly in order to retain large amounts of water, and as water is released from its structure, it returns to its original volume [3].The microcrack dimensions and ITZ thickness are generally greater in mortars containing CNC H than in those containing CNC S. Differences in CNC dimensions may contribute to this behavior, since CNC S and CNC H exhibit different characteristic sizes (149 ± 59 nm for CNC S and 266 ± 107 nm for CNC H). However, CNC surface chemistry may also play an important role because CNC S contains sulfate surface groups, whereas CNC H is predominantly hydroxyl-functionalized. At CNC concentrations above the optimum range, the increasing microcrack dimensions and ITZ thickness may also be associated with reduced CNC dispersion and increased nanocrystal–nanocrystal interactions.

Another interesting feature observed in the CNC-containing mortars is the formation of a ring- or shell-like region around some unhydrated cement particles. This feature may be associated with the adsorption of CNCs onto cement particles and a steric stabilization effect, as previously proposed for CNC-containing cementitious systems [34]. Such interfacial effects, together with the reduction in microcrack dimensions and ITZ thickness, may contribute to a more homogeneous cementitious microstructure and improved stress transfer between the cement paste and sand grains, thereby supporting the enhanced mechanical performance observed in the CNC-containing mortars.

### 3.3. Thermogravimetric Analysis (TGA)

**Figure 4:**
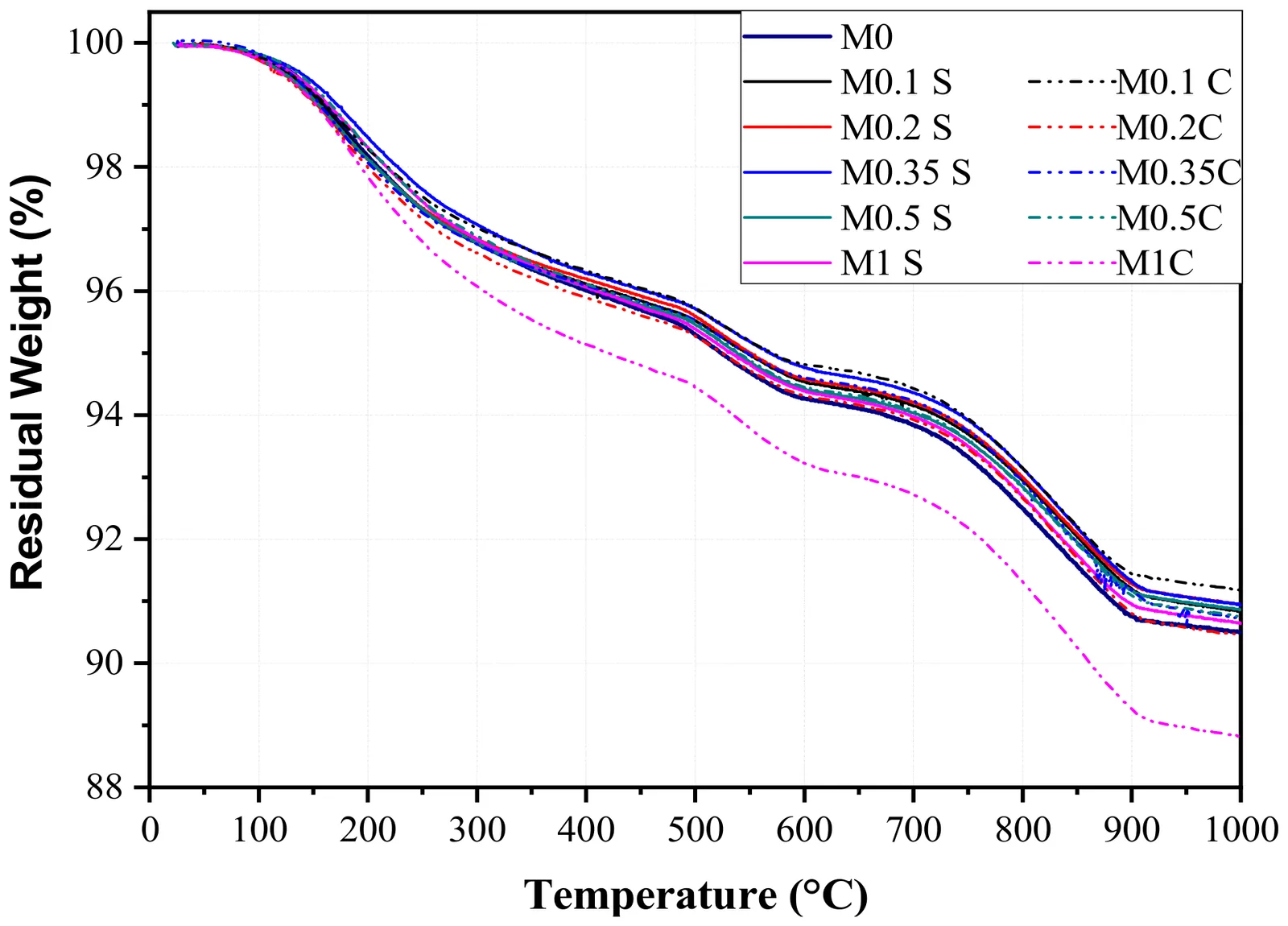
TGA curves of mortars containing CNC S and CNC H as a function of CNC content.

Figure 4 shows the TGA curves of the control mortar (M0) and mortars containing different concentrations of CNC S and CNC H. Overall, all samples exhibit similar thermal profiles, indicating that CNC incorporation does not substantially alter the main thermal decomposition stages of the cementitious matrix. Overall, the study variables generally exhibit similar trends in their temperature versus weight loss curves. When subdividing their curves into the standard ranges used in such studies, three general temperature regions can be distinguished:

(a) A common initial phase for all variables, characterized by a downward slope ranging from 90 to 400 °C, mainly associated with the loss of physically and chemically bound water from hydration products, with maximum acceleration of the slope increase occurring between 150 and 250 °C.
(b) In the temperature range between 500 and 600 °C, primarily related to the dehydroxylation of portlandite, no acceleration in the change in slope is observed, but there is an increase in the slope compared to the previous region.
(c) Temperature range between 700 °C and 1000 °C, mainly associated with the decomposition of carbonate-containing phases. In this case, the inflection point marking the start of the acceleration in the rate of increase is set at 750 °C.

The total mass losses of the different mortars were very similar. The control sample exhibited a total mass loss of 9.90 ± 0.024%, whereas the CNC-containing mortars showed comparable values. M0.35 S and M0.5 H, which exhibited the highest compressive strengths, both showed a total mass loss of approximately 9.88%. Therefore, total mass loss alone does not provide sufficient evidence to establish differences in thermal stability among the samples. Instead, the distribution of mass loss among the different temperature regions provides more useful information regarding changes in hydration products induced by CNC incorporation. The above applies in general, with the exception of sample M1 H (weight loss of 9.96%), which may have been due to structural and physical aspects of CNC use (overdosing of CNC caused it to agglomerate in the matrix, tending to adhere and precipitate), which create areas of high concentration that increase weight loss in the test [3].

The M0.35 S and M0.5 H mixtures (which exhibited better compressive strength) showed a weight loss of 9.88%. These values are lower than those reported by Metalssi et al. (2014) [59], in which cellulose ether was used (a difference of 1.56% compared to the control and with 0.1 to 2% replacement). Furthermore, Jiao et al. (2016) [33] added 0.4% CNF to the mortar, which showed a 1.7% weight loss compared to the control; and Gómez et al. (2013) [3] used a 3% CMC replacement in cement pastes (cement and water) and obtained a positive difference of 3.5% overall. Finally, there are studies in the literature that use sawdust and wood, which also show greater weight loss than the reference samples; these studies explain this effect by noting that the thermal degradation pattern is related to the pyrolysis of lignocellulosic materials [64].

**Figure 5:**
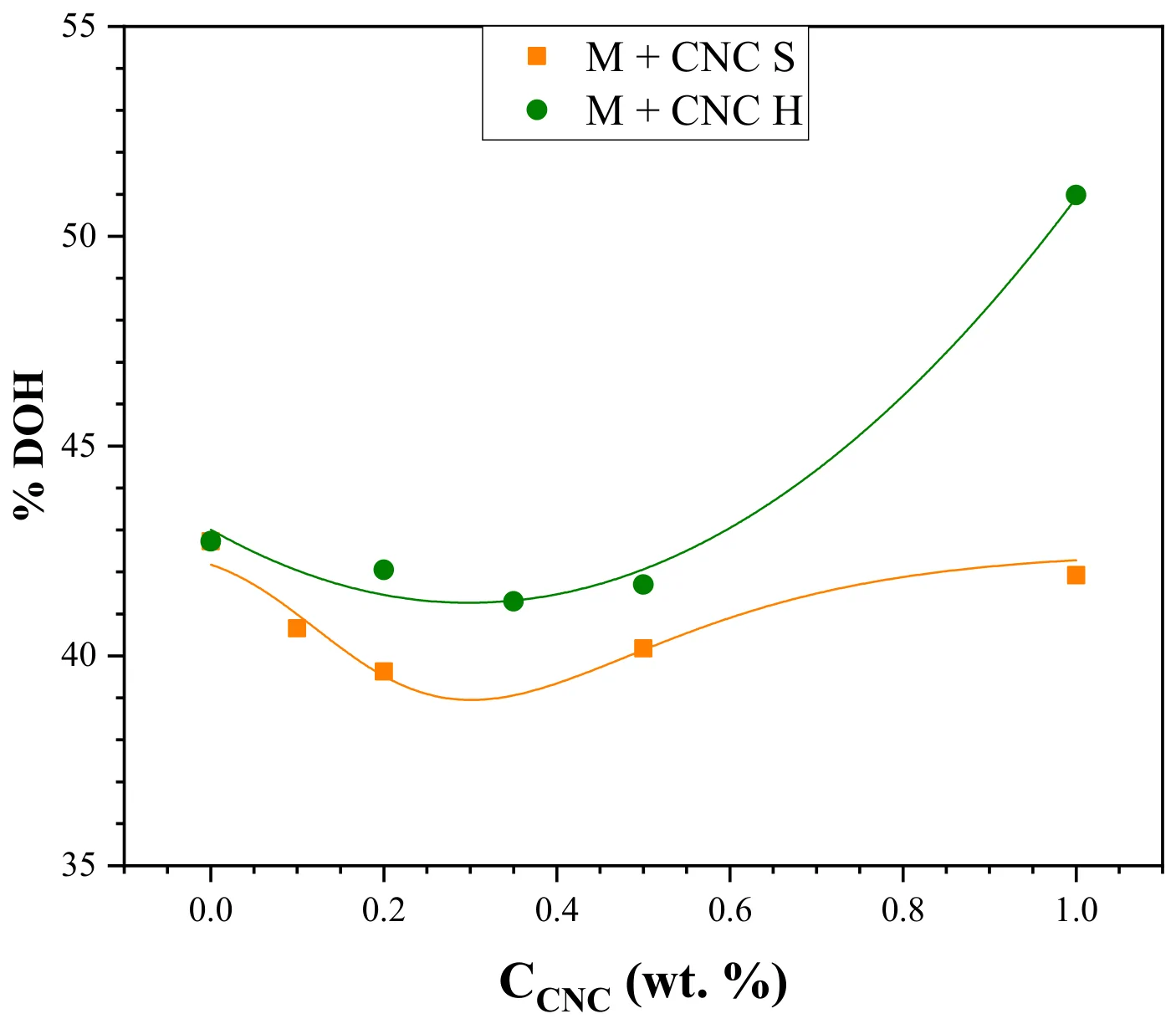
DOH of the mortars as a function of the amount of CNC.

At the same time, the degree of hydration (DOH) was estimated by measuring the total mass of chemically bound water (CBW) in the mortar matrices (determined by TGA). Figure 5 shows the percentage of the degree of hydration (% DOH) of the mortars, obtained using the method of Pane and Hansen (2005) [65], while Table S3 in the Supplementary Materials shows the values obtained. The mass loss between 140 and 1000 °C was used to estimate CBW, which was subsequently normalized to the final sample mass and related to the theoretical CBW content of fully hydrated cement (0.23 g g^−1^ cement) [34].

Figure 5 shows a graph of %DOH as a function of CNC concentration for mortars reinforced with CNC S and CNC H. The symbols represent the experimental data, while the lines represent the fit to Equation (1). Only small differences in DOH were observed among the samples, and these variations did not reproduce the trends observed for compressive or flexural strength. Therefore, the estimated DOH should be interpreted cautiously and suggests that the improvement in mechanical performance cannot be explained solely by changes in the overall degree of hydration. However, the theory governing the behavior of this type of matrix states that, in general, the presence of CNC will cause more water to react with the cement (in this case, the weight loss was lower and is attributed to the CNC). Another point is that there may be physical and structural effects that could “prevent” proportional behavior, such as the blocking or encapsulation of cement particles, until, at high doses of CNC, these effects are overcome and an increase in DOH occurs. One possible explanation for this increase is that CNCs help cement particles react more efficiently with water. This may be due to steric stabilization, which is the same mechanism observed in some types of water-reducing additives that disperse cement particles during mixing, resulting in finer and more uniform cement distributions [34].

CNCs have a high water-retention capacity, which may influence setting and cement hydration [3]. This behavior may favor the local formation of hydration products and contribute, together with microstructural and interfacial effects, to the improved mechanical performance [34,66]. Furthermore, CNCs are generally more useful than other types of nanomaterials in the cement matrix, such as cellulose nanofibers (CNFs), which have been found to cause aggregation and reduce the workability of such matrices [34]. According to Gómez et al. (2013) [3], microcrystalline cellulose particles also reduce the workability of fresh cement matrices and delay the hydration reaction. This is a significant difference compared to CNCs, since, because they are smaller, a larger volume of them is needed to cause agglomeration and impair the performance of these matrices; this gives CNCs an advantage over other nanomaterials.

CNCs have a high water-retention capacity and have been reported to influence cement hydration through water retention, nucleation, and dispersion effects [3,34,66]. However, only small variations in the estimated DOH were observed in the present study, and these variations did not reproduce the trends observed in the mechanical properties. Therefore, the improvement in mechanical performance cannot be attributed solely to an increase in the overall degree of hydration. Other factors, including CNC dispersion, surface chemistry, and the microstructural and interfacial modifications observed by SEM, may also contribute to the mechanical response. In particular, CNC S and CNC H differ in both dimensions and surface chemistry, with CNC S containing sulfate surface groups and CNC H being predominantly hydroxyl-functionalized. These differences may influence their interactions with cement particles and hydration products and, consequently, the resulting cementitious microstructure.

**Figure 6:**
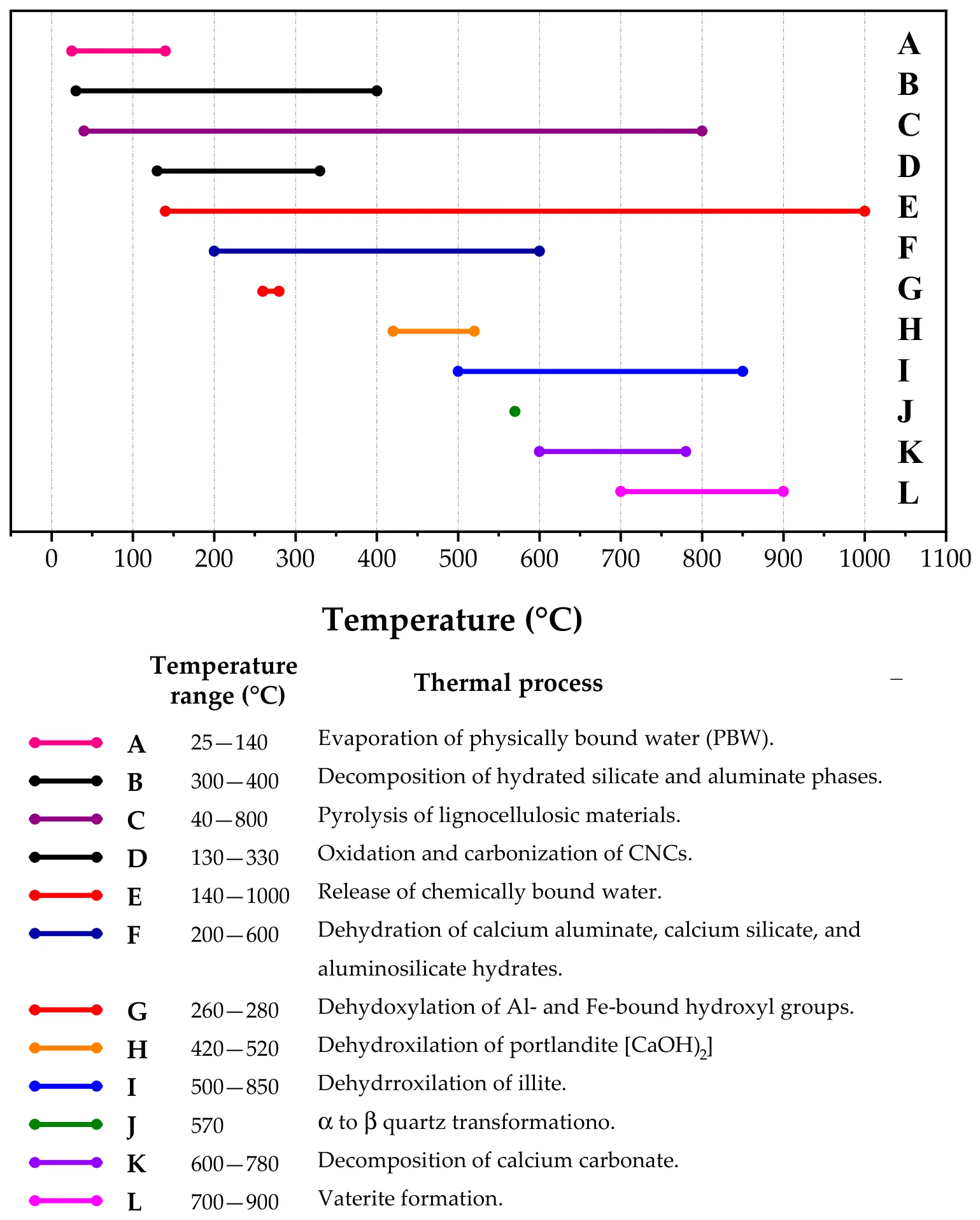
Reported temperature ranges associated with the main thermal processes of cementitious phases and cellulose nanocrystals [3,34,64,66,67,68,69,70].

Figure 6 shows the temperature ranges at which weight loss occurs for various compounds that make up the cementitious matrix and the CNCs [3,34,64,66,67,68,69,70]. The process can be broken down into the following simplified phases:

1. Loss of physically bound water (PBW);
2. Degradation of chemically bound water (CBW).

These phases make it possible to identify water losses and weight drops on the TGA curve.

**Figure 7:**
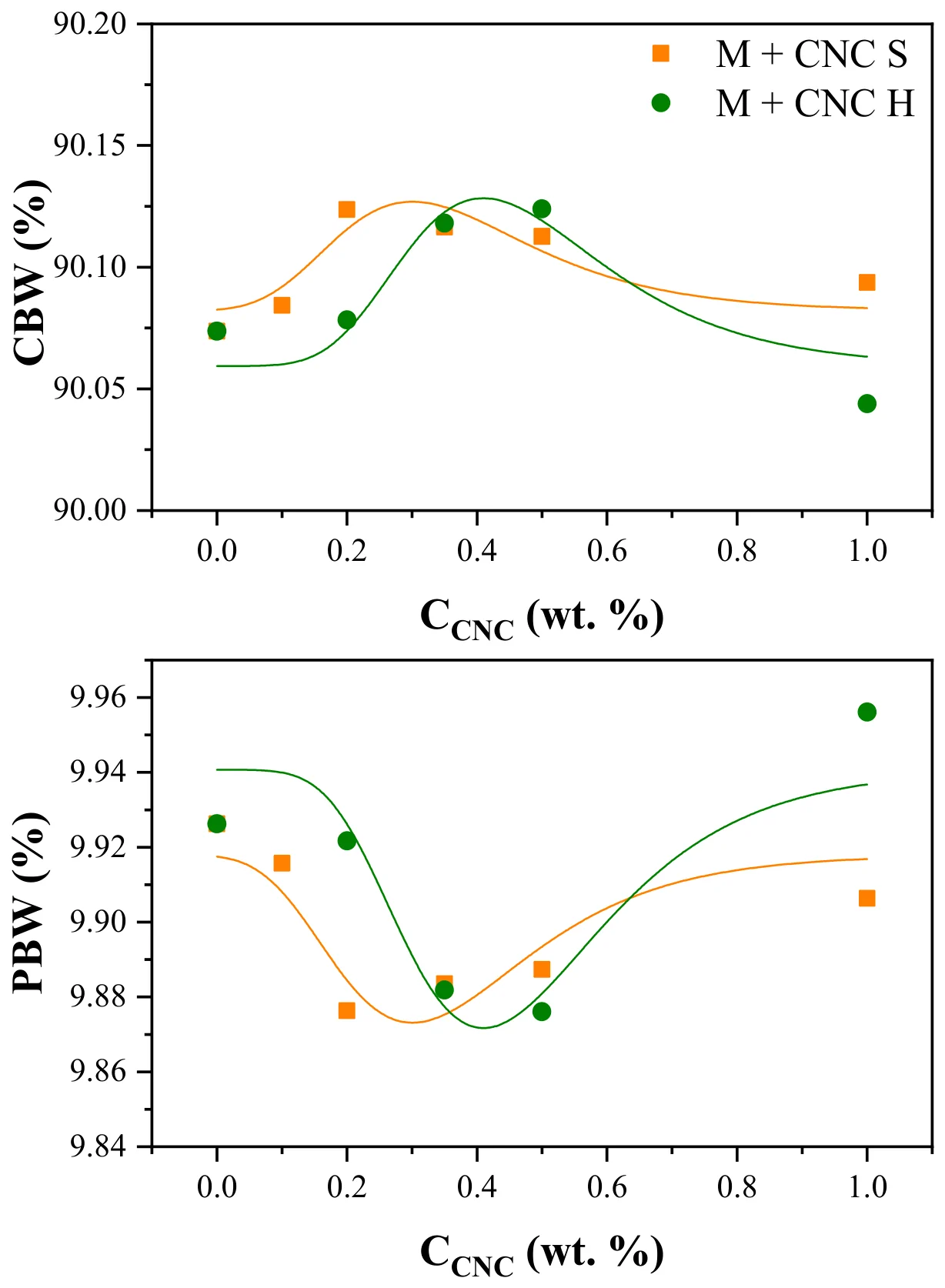
CBW and PBW for mortars with CNC S and CNC H.

Because the TGA curves largely overlap, the mass-loss contributions associated with physically bound water (PBW) and chemically bound water (CBW) were analyzed separately (determined by graphical integration using OriginPro 2021 from OriginLab. PBW was determined from the mass loss between 50 and 140 °C (the lower limit was set at 50 °C to minimize error, since the initial temperature varied for each measurement), whereas CBW was estimated from the mass loss between 140 and 1000 °C. The variations among samples were relatively small, approximately 0.25% for PBW and 0.43% for CBW; the obtained values are reported in Table S3 of the Supplemental Material. Nevertheless, both CNC types exhibited a similar non-monotonic trend and are shown in Figure 7 as a function of CNC concentration for CNC S and CNC H; the symbols represent the experimental results, while the curve represents the best fit to the experimental data (Equation (1)). CBW initially increased with CNC concentration, reached a maximum, and subsequently decreased, whereas PBW showed the opposite behavior. Interestingly, the maximum CBW contribution occurred within the CNC concentration range associated with the highest mechanical strengths.

This behavior suggests that, at appropriate concentrations, CNCs may favor the conversion of physically retained water into water associated with hydration products, consistent with their proposed water-retention and nucleation effects [65]. At higher CNC concentrations, reduced dispersion or CNC agglomeration may limit this effect.

The small difference between CNC S and CNC H in chemically bound water indicates that the negative charge carried by CNC S—while helping them repel one another—also allows them to form weak bonds with cement ions and ultimately retain more water than CNC H. Although the weight losses were not significantly different from those of the control sample, this confirmed that adding CNC to the mortar does not adversely affect its properties; on the contrary, it improves them.

**Figure 8:**
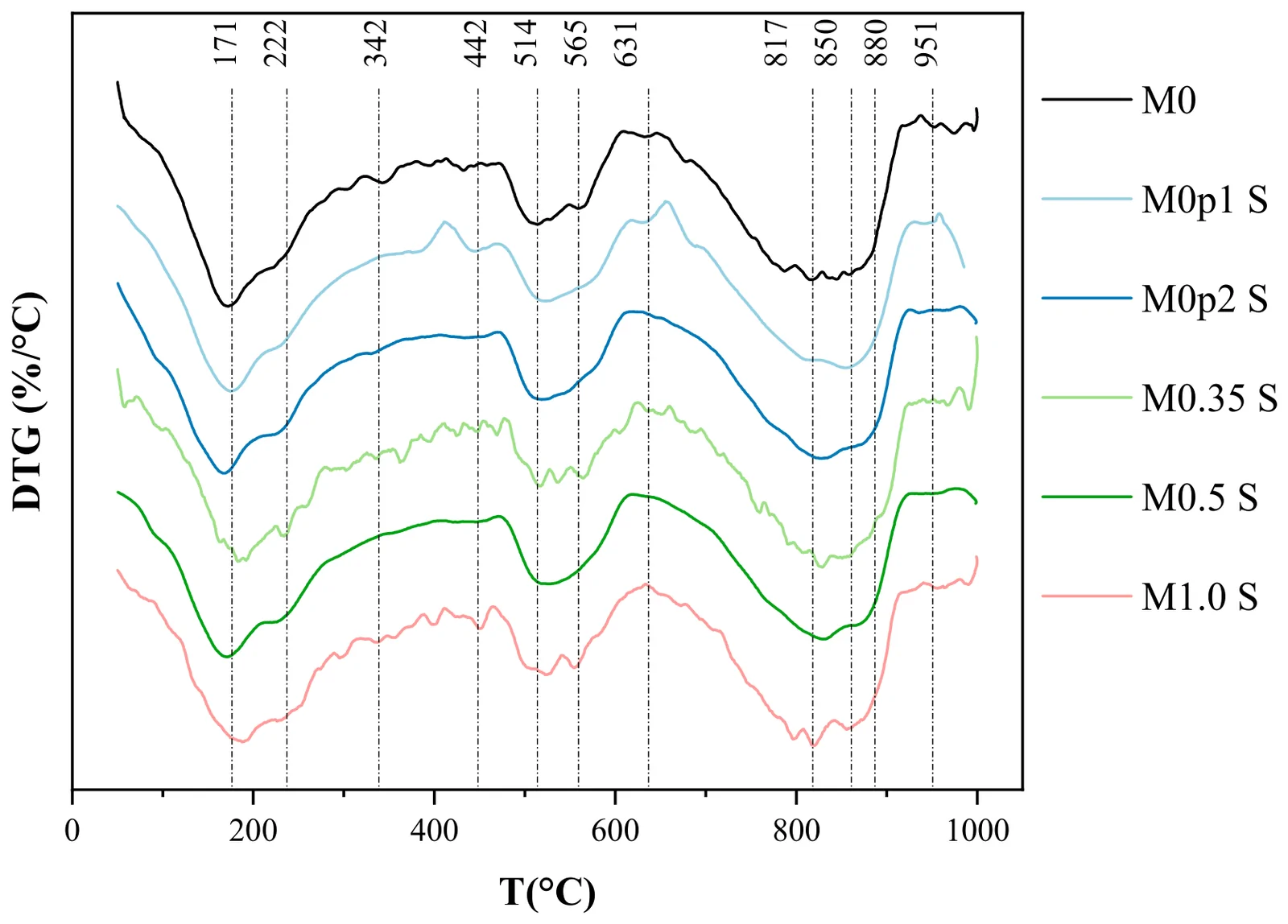
DTG curves of mortars containing CNC S as a function of temperature.

**Figure 9:**
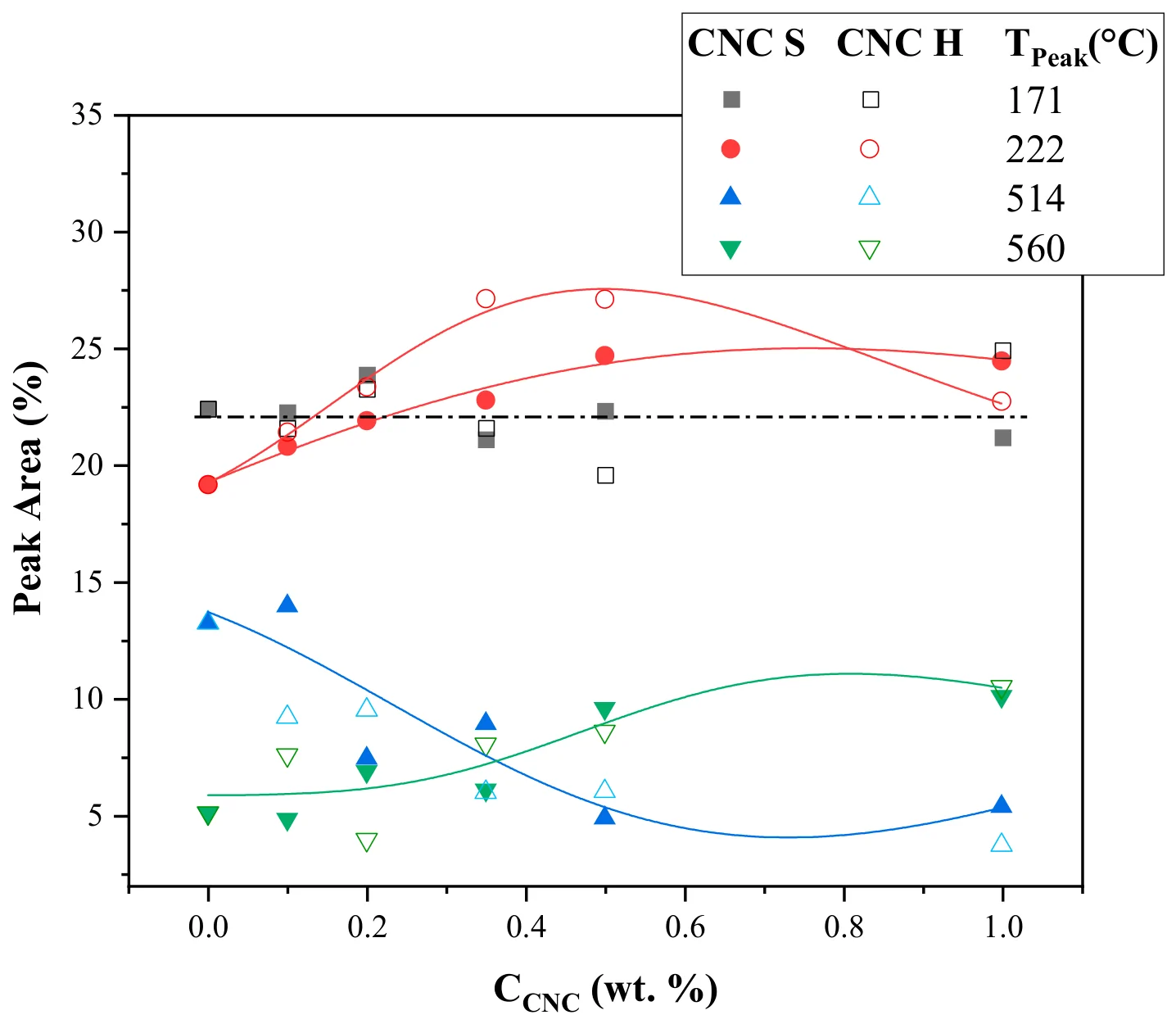
Peak area of the weight loss derivative as a function of CNC concentration for mortars containing CNC S and CNC H.

To further resolve the overlapping thermal events, derivative thermogravimetric (DTG) curves were calculated from the TGA data. Figure 8 shows representative DTG curves for mortars containing CNC S; similar analysis was performed for CNC H. Deconvolution of the DTG profiles revealed thermal events centered at approximately 171, 222, 514, 560, 818, and 844 °C (using deconvolution with Origin Lab’s Origin 21). Figure 9 compares the areas of the events at 171, 222, 514, and 560 °C as a function of CNC concentration.

The peak at 171 °C, representing 22.2 ± 1.4%, tends to remain constant with random variation relative to the CNC concentration; it may be associated with the release of weakly bound water from hydrated phases. In contrast, the area of the event at approximately 222 °C increased with CNC incorporation. This behavior may reflect an increased contribution from chemically bound water associated with hydrated products, including C-S-H and hydrated aluminate phases [71], and is consistent with a possible influence of CNC on hydration and nucleation processes. This behavior is consistent with a possible influence of CNCs on the physicochemical environment of hydrated phases and/or nucleation processes; however, this thermal contribution alone does not demonstrate an increase in the overall degree of hydration.

The peak at 514 °C shows a decrease in area with increasing CNC concentration, which can be attributed to the dehydroxylation of portlandite, suggesting that there is less portlandite in the system because CNCs promote the formation of C-S-H gel, either through more efficient hydration or through interaction between the CNC surface and calcium ions, which is corroborated by the observed increase in the peak areas at 222 °C [71,72]. However, the increase in peak area at 560 °C and the nonlinear behavior observed in the peak areas at 844 °C suggest that the area diminution at the peak of 514 °C should not necessarily be interpreted as a direct reduction in the amount of portlandite [71]. Rather, the redistribution of the DTG contributions in this temperature region suggests changes in the thermal environment and/or organization of calcium-containing phases. At higher concentrations, CNCs may alter the microstructure, promoting the formation of carbonate phases or leading to agglomeration, rather than simply causing a monotonic increase or decrease in a single hydration product [71,72].

Overall, the TGA/DTG results indicate that CNC incorporation has only a minor effect on total mass loss but modifies the relative contributions of individual thermal events associated with hydrated and carbonated phases. These changes depend on CNC concentration and surface chemistry. To determine whether these thermal differences are accompanied by changes in the crystalline phases of the cementitious matrix, the mortars were subsequently analyzed by XRD.

### 3.4. X-Ray Diffraction (XRD)

**Figure 10:**
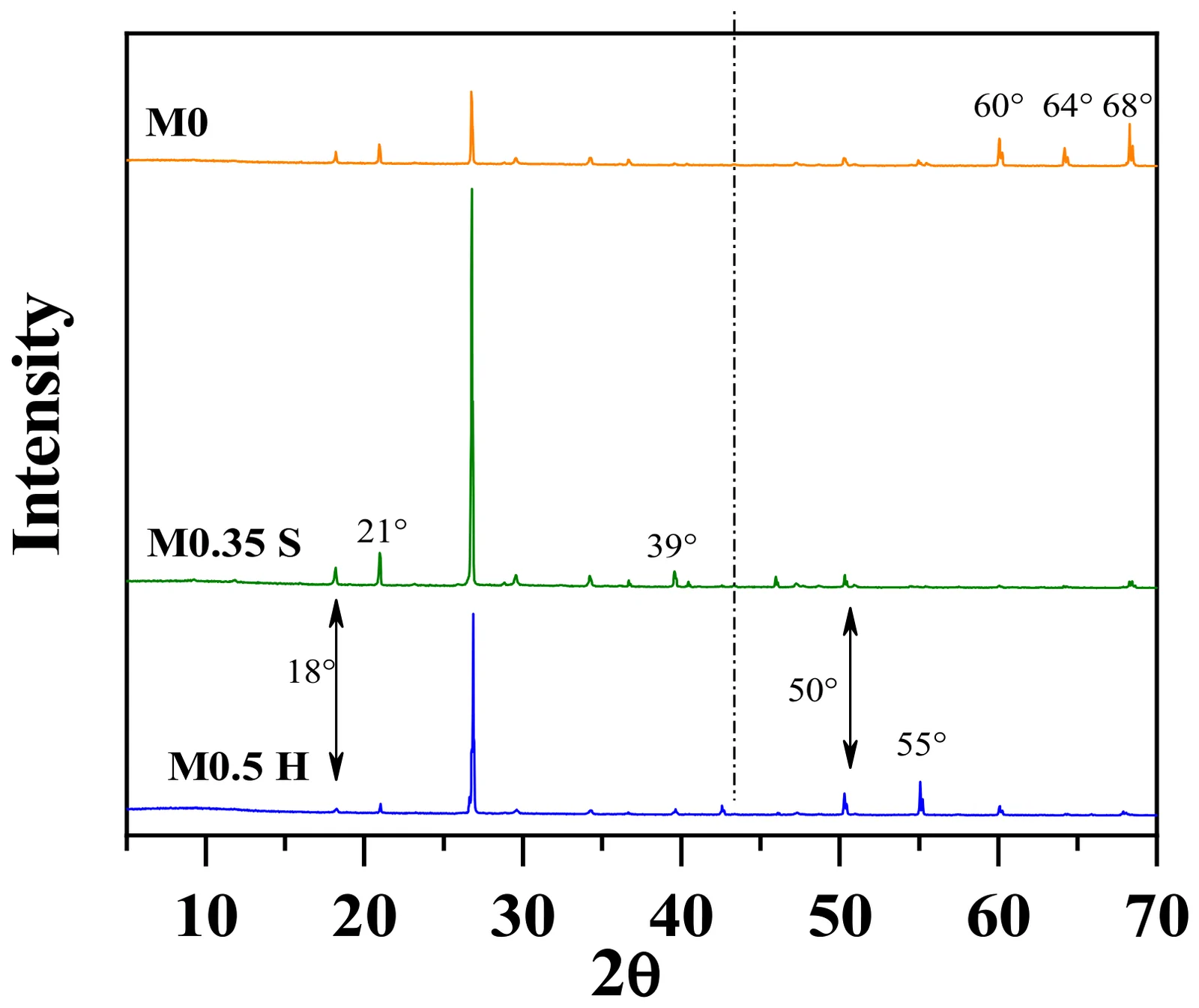
Diffractograms of the mortars containing CNC S (MS), CNC H (MH), and the blank reference (M0).

Figure 10 shows the XRD patterns of the control mortar (M0) and the mortars containing 0.35 wt.% CNC S (M0.35 S) and 0.5 wt.% CNC H (M0.5 H). The three diffractograms exhibit similar profiles, with the same main crystalline phases but differences in relative peak intensities. The most intense reflection is observed at approximately 26° 2θ and is assigned to quartz (Q, SiO_2_), which originates predominantly from the siliceous sand. Additional quartz reflections are observed at approximately 22°, 39°, 43°, 60°, and 68° 2θ. Portlandite (P, Ca(OH)_2_) is identified by reflections at approximately 18°, 37°, 47°, and 51° 2θ, whereas reflections associated with residual anhydrous cement phases are observed at approximately 29° for tricalcium silicate (C_3_S, alite) and 33° for dicalcium silicate (C_2_S, belite). A reflection at approximately 5° is assigned to muscovite [M, KAl_2_(AlSi_3_O_10_)(OH)_2_] [64,70,73,74].

No additional crystalline phases were detected after CNC incorporation, indicating that the overall crystalline phase composition of the mortars remained essentially unchanged. However, differences in the relative intensities of some portlandite reflections were observed in M0 compared to M0.35 S and M0.5 H. These variations suggest that CNC incorporation influences the development and/or crystalline organization of hydration products [59,75]. Nevertheless, because conventional XRD measurements are semi-quantitative and peak intensities may also be affected by preferred orientation, crystallite characteristics, and sample preparation, these differences should be interpreted with caution [60].

Two portlandite reflections are particularly noteworthy. The reflection at approximately 18° 2θ becomes more intense in the mortar containing CNC S, whereas the reflection at approximately 51° 2θ shows a comparatively greater increase for CNC H. Since both reflections correspond to portlandite, these differences do not necessarily indicate the formation of distinct crystalline phases. Rather, they may reflect differences in preferred crystallographic orientation, crystallite size or degree of ordering of portlandite formed in the presence of the two CNC types.

The different behavior of CNC S and CNC H may be related to their surface chemistry. The sulfate groups present on CNC S can interact with Ca^2+^ ions and may provide preferential sites for the nucleation and growth of calcium-containing hydration products. In contrast, CNC H lacks significant surface sulfate groups and contains predominantly hydroxyl-functionalized surfaces; therefore, its influence on hydration may be associated mainly with water retention, surface nucleation, and hydrogen-bonding interactions. These different interfacial environments may affect the growth and organization of portlandite crystals without necessarily changing the overall crystalline phase composition of the mortar.

This interpretation is consistent with the DTG results (Figure 9), where the contribution centered at approximately 514 °C decreases while that around 560 °C increases with CNC incorporation. Rather than indicating a simple increase or decrease in the total portlandite content, this redistribution of thermal contributions may reflect changes in the physicochemical environment, organization, or thermal stability of calcium-containing phases. The combined XRD and TGA results therefore suggest that CNC incorporation modifies the organization of hydration products and that the response depends, at least in part, on CNC surface chemistry.

Importantly, these results indicate that sulfate groups are not a prerequisite for CNC reinforcement, although they may contribute to the differences observed between the two CNC types. Furthermore, the relative mechanical performance of CNC S and CNC H varied with CNC concentration and loading mode, with a crossover in their relative response occurring at approximately 0.5 wt.%. This concentration-dependent behavior further suggests that the mechanical response cannot be attributed solely to the sulfate groups of CNC S, but rather to the combined effects of CNC concentration, dimensions, surface chemistry, dispersion, and CNC–matrix interactions. Although sulfate-related contributions may be present, their specific effects on ettringite formation or early-age hydration cannot be conclusively established from the present data.

### 3.5. Infrared Spectroscopy (FTIR)

**Figure 11:**
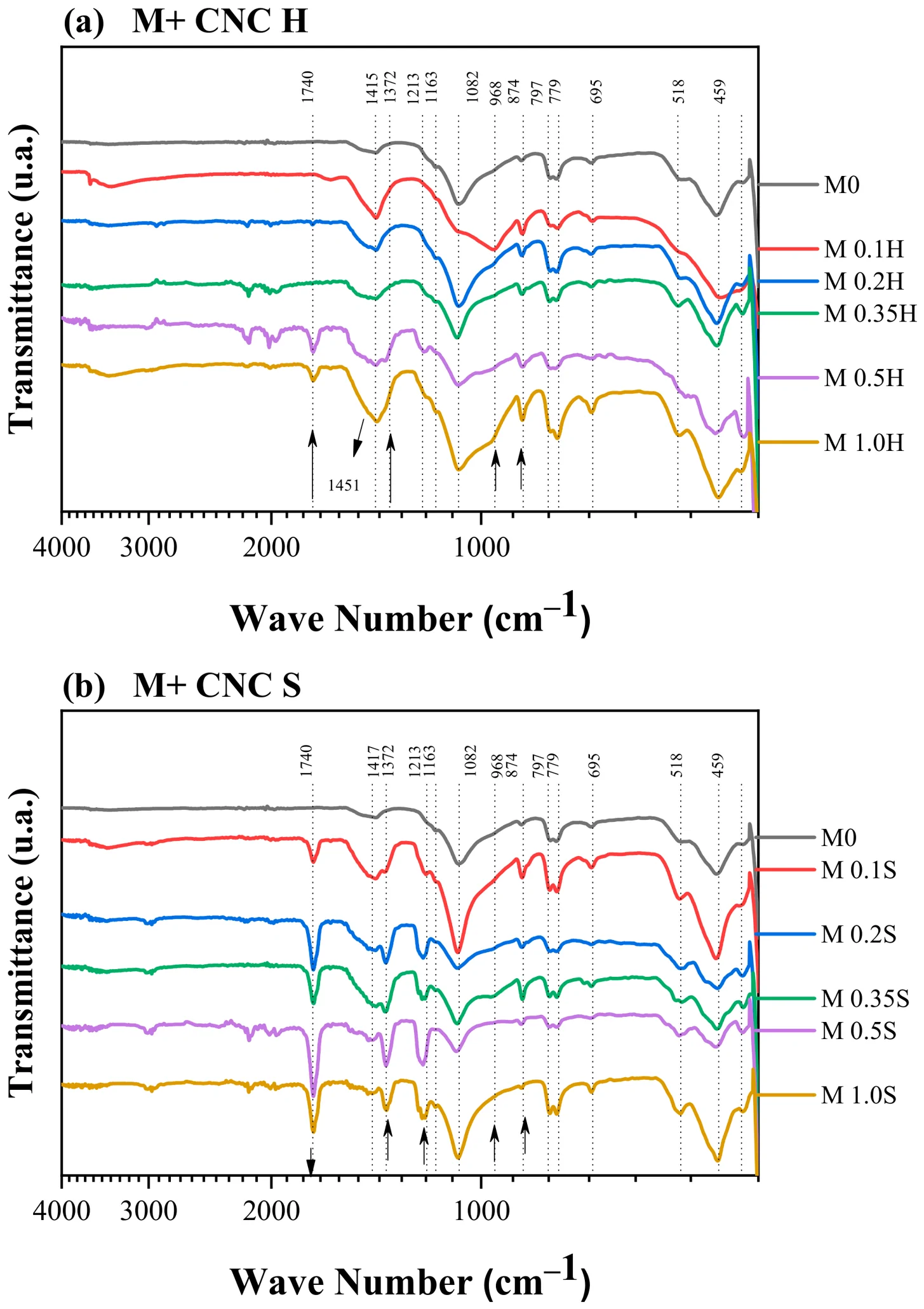
FTIR spectra of mortars containing CNC H and CNC S as a function of CNC concentration.

Figure 11 shows the FTIR-ATR spectra of mortars containing (a) CNC S and (b) CNC H at different concentrations, compared with the control mortar (M0). The characteristic absorption bands of cementitious materials can be identified in several spectral regions [76]:

(a) In the 400–800 cm^−1^ region, several bands associated with silicate phases are observed. The band at approximately 475 cm^−1^ is attributed to Si–O bending vibrations in SiO_2_ [76,77,78,79,80], whereas the band at approximately 695 cm^−1^ is associated with symmetric Si–O vibrations in SiO_4_ units [76,79]. Bands at approximately 459 and 518 cm^−1^ have been associated with Al–O/Si–O vibrations [79], while the doublet at 779 and 797 cm^−1^ is characteristic of crystalline α-quartz [77,80,81]. Furthermore, the doublet indicates that the quartz in the silica sand is crystalline in the α-phase.
(b) In the range from 900 to 1000 cm^−1^, hydration products such as C–S–H gel appear in concrete. The band at approximately 968 cm^−1^ is commonly assigned to Si–O stretching vibrations in C–S–H-type hydration products [78,79,82,83,84,85,86,87]. The band around 874 cm^−1^, together with the intense absorption around 1415 cm^−1^, is mainly associated with carbonate species resulting from carbonation of the cementitious matrix, attributed to the bending and stretching vibrations, respectively, in the plane of the C–O and C–H bonds resulting from the reaction between Ca(OH)_2_ and CO_2_ in the air [88]. On the other hand, Kocak reports that the peak at 871 corresponds to the Si–O bond [79].
(c) At approximately 1200 cm^−1^, the silicates and aluminates formed during cement hydration appear [76]. It has been reported that the bands at 1120 cm^−1^ and 1140 cm^−1^ are due to the S–O vibrations of the sulfate present in ettringite [Ca_6_Al_2_(SO_4_)_3_(OH)_12_·26H_2_O] [89], which is a hydration product of tricalcium aluminate (C3A) with calcium sulfate (CaSO_4_), both of which are present in cement [67,86,87,88,89,90]. However, in these spectra, these bands overlap with the CaCO_3_ band at 1083 cm^−1^, which exhibits a shoulder at 1167 cm^−1^—also attributed to silica sand—along with the signals at 475 and 779–797 cm^−1^ [77,80,87,91].
(d) The bands observed around 1400–1450 cm^−1^ are primarily associated with C–O stretching vibrations of carbonate species, and peaks at 1415 and 1451 cm^−1^ can be observed. Similar bands have been reported at 1420 [79], 1429 [78], and 1431 [86].
(e) The sharp band at approximately 3646 cm^−1^ is assigned to O–H stretching in portlandite, Ca(OH)_2_ [78,88,90,92], whereas the broad absorption extending approximately from 3100 to 3700 cm^−1^ is associated predominantly with hydrogen-bonded O–H groups and adsorbed or bound water in the hydrated cementitious matrix.

Although CNC incorporation does not produce major changes in the overall FTIR profiles, differences in the relative intensity and shape of several bands are evident between mortars containing CNC S and CNC H, particularly around 3400, 1740, 1417, 1372, 1213, 1082, and 968 cm^−1^. These differences suggest that CNC surface chemistry influences the local chemical environment of the cement hydration products.

Analyzing the spectra from left to right, a very small peak at 3646 cm^−1^ is visible in all of them, corresponding to the hydroxyl group in Ca(OH)_2_ [78,88,90,92]. The broad O–H region is more pronounced in some CNC-containing mortars, particularly those containing CNC H. The most notable finding here relates to the reduction in the hydroxyl group peak in the M + CNC S and M + CNC H spectra, compared to the CNC S and CNC H spectra [45], which may indicate the formation of bonds between the hydroxyl groups of the CNC and the cement hydration products. However, direct comparison with the spectra of isolated CNC must be interpreted cautiously because of the low CNC concentration in the mortar. Changes in the position, shape, and relative intensity of this region are nevertheless consistent with modification of the hydrogen-bonding environment after CNC incorporation and may reflect interactions between CNC hydroxyl groups, water, and cement hydration products. Potential implications for charge transfer between CNC particles and hydration products have been reported, which affect the mechanical behavior of mortars [60].

A weak band around 1740 cm^−1^ is observed in the CNC-containing mortars, particularly in mortars containing CNC S and at the higher concentrations of CNC H. This region is commonly associated with C=O stretching vibrations [93]; neither the cement, its hydration products, nor the CNC contains this bond. Therefore, a hypothesis has been proposed that interactions exist between OH or SO_3_ groups in the CNC and the components that make up the mortar matrix, likely generating unidentified species containing carbonyl (C=O) groups [81]. The appearance and concentration-dependent intensity of this band indicate a modification of the chemical environment associated with carbonyl-containing species after CNC incorporation. However, FTIR alone does not allow unambiguous identification of the species responsible for this absorption or demonstrate the formation of new covalent bonds between CNC and the cementitious matrix. The more pronounced contribution observed for CNC S suggests that the different surface chemistry of sulfated CNC may influence these interactions.

A further difference between CNC S- and CNC H-containing mortars is observed in the 1350–1450 cm^−1^ region. For CNC H, a broad and intense band centered around 1415 cm^−1^ is observed, with the contribution near 1372 cm^−1^ appearing mainly as a shoulder. In contrast, mortars containing CNC S exhibit a better-resolved contribution near 1372 cm^−1^ [94,95]. As previously mentioned, the bands around 1410 cm^−1^ in mortars are due to carbonates, which originate from the cement raw materials and carbonation during storage or use. For CNC S, this can be attributed to vibrations associated with sulfate groups or with carbonates that are less crystalline or modified by interaction with Ca^2+^; the surface sulfate groups can coordinate with calcium ions, creating more well-defined chemical environments and causing this signal to be better resolved [96]. For CNC-H, which does not contain any significant surface sulfate groups, the 1372 cm^−1^ signal is superimposed on the 1417 cm^−1^ signal, which is typical of carbonates, particularly CO_3_^2−^ from calcite or matrix carbonation [34]. This suggests that in CNC H, the contribution of calcium carbonates is more dominant, whereas in CNC S, there is an additional contribution from sulfate or carbonate groups in different environments.

The band near 968 cm^−1^, assigned mainly to Si–O stretching vibrations in C–S–H-type hydration products, is more clearly resolved in mortars containing CNC H. This may indicate differences in the local silicate environment and/or organization of C–S–H-type products in the presence of CNC H. Such behavior is consistent with the proposed ability of hydroxyl-rich CNC surfaces to retain water and provide nucleation sites for hydration products [97,98]. This is consistent with the idea that CNC-H physically interacts with the matrix via hydrogen bonding. In mortars containing CNC S, the contribution near 968 cm^−1^ appears mainly as a shoulder of the more intense band centered around 1083 cm^−1^. The sulfate groups present on CNC S may contribute to the complex overlapping vibrations in the 1000–1200 cm^−1^ region, where sulfate- and silicate-related bands coexist. Consequently, the C–S–H-associated contribution may be less clearly resolved in CNC S-containing mortars [98]. These differences are qualitatively consistent with the DTG results, particularly the changes observed in the thermal event around 222 °C, where greater weight loss is observed for CNC H than for CNC S, associated with chemically bound water in hydrated phases. Taken together, FTIR and DTG suggest that CNC S and CNC H influence the hydration environment differently, although neither technique alone allows quantitative determination of the amount of C–S–H formed.

Taken together, these results suggest that CNC S and CNC H modify the local chemical environment of cement hydration products in different ways. The differences are consistent with their distinct surface chemistries: CNC S contains sulfate groups in addition to hydroxyl groups, whereas CNC H is predominantly hydroxyl-functionalized. Thus, both CNC types may participate in interfacial interactions with the cementitious matrix, but the relative contributions of sulfate-mediated interactions, hydrogen bonding, water retention, and surface nucleation are expected to differ.

These spectroscopic differences, together with the microstructural, thermal, and XRD results, support the hypothesis that the mechanical reinforcement provided by CNCs arises not only from their nanoscale dimensions but also from concentration-dependent interfacial interactions governed by their surface chemistry.

The present study demonstrates improvements in mechanical performance and microstructural refinement at relatively low CNC contents; however, these results should not be interpreted as direct evidence of improved long-term durability. Properties such as drying shrinkage, water absorption and sorptivity, chloride penetration, carbonation resistance, freeze–thaw behavior, and long-term dimensional stability were not evaluated. These properties may be affected by CNC-induced changes in pore structure, water retention, dispersion, and the cement–aggregate interfacial region. Therefore, systematic long-term durability studies are required to determine whether the mechanical and microstructural improvements observed at the optimum CNC contents are maintained under environmental exposure and service conditions.

## 4. Conclusions

Incorporating cellulose nanocrystals from Agave tequilana bagasse improved the mechanical performance of Portland cement mortars at relatively low CNC contents. The highest compressive strengths were obtained at 0.35 wt.% CNC S and 0.5 wt.% CNC H, corresponding to increases of approximately 35% and 34%, respectively, relative to the control mortar. Flexural strength also increased, reaching improvements of approximately 27% for CNC S and 20% for CNC H at 0.5 wt.% CNC.

SEM analysis showed that CNC incorporation modified the mortar microstructure, reducing the size and number of microcracks and decreasing the thickness of the interfacial transition zone near the CNC concentrations associated with maximum mechanical strength. These observations suggest that, at appropriate concentrations, CNCs contribute to a more homogeneous cementitious microstructure and improved stress transfer at the cement paste–sand interface.

The TGA/DTG results showed only minor differences in total mass loss among the mortars but revealed changes in the relative contributions of hydrated and carbonated phases. In particular, the increase in the thermal event near 222 °C and the redistribution of the contributions around 514–560 °C suggest that CNC incorporation modifies the physicochemical environment of cement hydration products rather than simply increasing or decreasing a single phase.

XRD analysis showed that CNC incorporation did not generate additional crystalline phases, although changes in the relative intensities of portlandite reflections were observed. These differences suggest that CNC S and CNC H may influence the organization and growth of calcium-containing hydration products differently, likely because of differences in their surface chemistry.

FTIR analysis further supported this interpretation. Mortars containing CNC H exhibited a more clearly resolved band near 968 cm^−1^, associated with Si–O vibrations in C-S-H-type hydration products, whereas in CNC S-containing mortars this contribution appeared mainly as a shoulder of the more intense band near 1083 cm^−1^. Differences in the 1372–1415 cm^−1^ region also indicate that sulfate-functionalized CNC S and predominantly hydroxyl-functionalized CNC H modify the local chemical environment of the cementitious matrix in different ways.

Taken together, the mechanical, microstructural, thermal, XRD, and FTIR results indicate that CNC reinforcement depends not only on nanocrystal size and concentration but also on surface chemistry. CNC S and CNC H achieve comparable mechanical improvements through interfacial environments that are not necessarily identical. CNC derived from Agave tequilana bagasse therefore represents a promising strategy for valorizing agroindustrial waste while improving the performance of cementitious materials and contributing to more sustainable construction.

## Figures and Tables

**Table 1:** Physicochemical characteristics of CNC S and CNC H used in this study [45].

Property	CNC S	CNC H
Hydrolysis	H_2_SO_4_	HCl
Crystallinity (%)	91.3	90.1
Hydrodynamic diameter (nm)	222 ± 13	329 ± 6
Length (nm)	149 ± 59	266 ± 107
Height (nm)	~9	~9
L/H	16 ± 6	31 ± 12
W/H	4.2 ± 1.3	10 ± 1.7
Surface sulfate groups	166 mmol kg^−1^	Not significant
Main surface functionality	–OH/sulfate ester	predominantly –OH

**Table 2:** Values obtained by fitting the data from Figure 1c,d to Equation (1).

	M + CNC H	M + CNC S	Average	M + CNC H	M + CNC S	Average
**y** **_0_**	22.2 ± 1.41	25.84 ± 0.63	22.9	5.63	5.63	5.63
**xc**	0.61 ± 0.03	0.35	0.55 ± 0.03	0.71 ± 0.026	0.34 ± 0.036	0.62 ± 0.04
**w**	0.38 ± 0.08	0.14 ± 0.05	0.35 ± 0.05	0.34 ± 0.03	0.25 ± 0.05	0.37 ± 0.04
***A***	8.54 ± 1.29	5.10	7.52 ± 0.94	1.96 ± 0.14	1.33 ± 0.17	1.36 ± 0.09
***R*** **^2^**	0.9468	0.5754	0.8825	0.8415	0.9853	0.96038

## Data Availability

The original contributions presented in this study are included in the article/Supplementary Materials. Further inquiries can be directed to the corresponding author.

## Supplementary Materials

The following supporting information can be downloaded at https://www.mdpi.com/article/10.3390/nano16181142/s1. Table S1. Compressive and Flexural Strength of mortars with CNC S, CNC H, average and statistical analyses. Table S2. Average microcracks and ITZ sizes of the mortars. Table S3. Degree of hydration (DOH), physically bound water (PBW), and chemically bound water (CBW) obtained from TGA analysis.
