# Supplementary material for: Cellulose Nanocrystals Obtained from Agave tequilana Weber Bagasse as Reinforcing Agents for CPC-30R Portland Cement Mortar
Source: Nanomaterials (Basel). 2026 Sep 11;16(18):1142. doi: 10.3390/nano16181142 (PMC13610728; doi:10.3390/nano16181142)
Supplement: Supplementary file 1 [file nanomaterials-16-01142-s001.zip › nanomaterials-4552901-supplementary.pdf]

Support Material

Table S1. Compressive and Flexural Strength of mortars with CNC S, CNC H, average and statistical analyses.

C_{CNC} (wt. %)	Sample codes		Compressive Strength (MPa)		Flexural Strength (MPa)		Average (MPa)	
	**CNC S**	**CNC H**	**CNC S**	**CNC H**	**CNC S**	**CNC H**	**Comp Str**	**Flex Str**
**0**	M0	M0	22.9 ±1.7	22.9 ± 1.7	5.6 ± 0.7	5.6 ± 0.7	22.9 ± 1.5	5.6 ± 0.7
**0.1**	M0.1 S	M0.1 H	25.6 ± 1.7	30.0 ± 1.6	5.6 ± 0.5	6.4 ± 0.5	27.8 ± 2.8	6.0 ± 0.2
**0.2**	M0.2 S	M0.2 H	28.2 ± 1.5	26.0 ± 2	7.1 ± 0.4	6.7 ± 0.5	27.1 ± 2.2	6.9 ± 0.2
**0.35**	M0.35 S	M0.35 H	**30.9 ± 1.7**	27.9 ± 0.5	6.5 ± 0.5	--	29.4 ± 1.9	6.5 ± 0.2
**0.5**	M0.5 S	M0.5 H	27.9 ±1.8	**30.7 ± 2.6**	**7.1 ± 0.4**	**6.7 ± 0.3**	29.3 ± 2.6	6.9 ± 0.3
**1**	M1 S	M1 H	26.5 ± 0.6	28.0 ±0.6	7.1 ± 0.4	6.0 ± 0.5	27.2 ± 1.0	6.5 ± 0.3
**p_A value**			p=0.2307, **p>0.05**		p=0.8476, **p>0.05**			
**p_B value**			p=4.6x10^{-8}, p<0.05		p=3.4x10^{-5}, p<0.05			
**p_{A-B} value**			p=4.8x10^{-4}, p<0.05		p=1.6x10^{-2}, p<0.05			

±Standard deviation; A Factor: CNC type (CNC S or CNC H); B Factor: CNC concentration (0, 0.1, 0.2, 0.35, 0.5 and 1 wt. %). pA, pB, pAB: p values for A, B factors, and interaction between A and B, respectively. p < 0.05: A, B, or interactions between A and B are significantly different, p>0.05: no significant.

Table S2. Average microcracks and ITZ sizes of the mortars.

C_{CNC}	Average Microcrack width (μm)		Average Microcrack length (μm)		ITZ Average width (μm)	
	**CNC S**	**CNC H**	**CNC S**	**CNC H**	**CNC S**	**CNC H**
**0**	1.6 ± 0.3	1.6 ± 0.3	50 ± 9	50 ± 9	4 ± 2	4 ± 2
**0.1**	2.3 ± 0.5	1.2 ± 0.5	33 ± 8	19 ± 6	0.95 ± 0.25	1.5 ± 0.3
**0.2**	0.96 ± 0.4	1.3 ± 0.3	14 ± 16	22 ± 6	1.2 ± 0.6	1.8 ± 0.4
**0.35**	1.1 ± 0.2	1.0 ± 0.4	15 ± 4	29 ± 6	1.2 ± 0.3	1.3 ± 0.3
**0.5**	2.00 ± 0.5	1.2 ± 0.6	14 ± 5	22 ± 8	1.9 ± 0.3	2 ± 0.4
**1**	1.4 ± 0.5	1.5 ± 0.6	39 ± 19	35 ± 18	1.7 ± 0.7	1.6 ± 0.7

Table S3. Degree of hydration (DOH), physically bound water (PBW), and chemically bound water (CBW) obtained from TGA analysis.

C_{CNC} (wt. %)	DOH (%)		PBW (%)		CBW	
	**CNC S**	**CNC H**	**CNC S**	**CNC H**	**CNC S**	**CNC H**
**0**	42.7	42.7	9.926	9.926	90.074	90.074
**0.1**	40.6	39.1	9.917	9.870	90.084	91.130
**0.2**	39.6	42.1	9.876	9.921	90.124	90.078
**0.35**	40.9	41.3	9.883	9.882	90.116	90.118
**0.5**	40.2	41.7	9.887	9.876	90.113	90.124
**1**	41.9	51.0	9.906	9.956	90.094	90.044
